# Evolocumab effectiveness in the real-world setting: Austrian data from the pan-European observational HEYMANS study

**DOI:** 10.1007/s00508-023-02245-w

**Published:** 2023-07-31

**Authors:** Christoph Ebenbichler, Heinz Drexel, Ursula Hanusch, Hermann Toplak, Nafeesa N. Dhalwani, Ian Bridges, Robert Hoelzl, Margit Hemetsberger, Kausik K. Ray

**Affiliations:** 1 Department of Internal Medicine I, Medical University Innsbruck, Anichstraße 35, 6020 Innsbruck, Austria; 2 Vorarlberg Institute for Vascular Investigation & Treatment (VIVIT) at Feldkirch Academic Teaching Hospital, Feldkirch, Austria; 3 Center for Clinical Studies, Dr. Hanusch GmbH, Vienna, Austria; 4 Department of Internal Medicine, Medical University Graz, Graz, Austria; 5 Amgen Inc., Thousand Oaks, CA USA; 6 Amgen Ltd., Uxbridge, UK; 7 Amgen GmbH, Vienna, Austria; 8 Hemetsberger medical services, Vienna, Austria; 9 Imperial Centre for Cardiovascular Disease Prevention and Imperial Clinical Trials Unit, Imperial College London, London, UK

**Keywords:** Evolocumab, PCSK9i, LDL‑C, Registry, Guidelines, Cardiovascular risk

## Abstract

**Background:**

This real-world study examined clinical characteristics and dyslipidemia management among patients initiating evolocumab across 12 European countries. Austrian data are reported.

**Methods:**

Data of consenting adults were collected for ≤ 6 months prior to evolocumab initiation (baseline) and ≤ 30 months post-initiation. Patient characteristics, lipid lowering therapy (LLT, i.e. statin and/or ezetimibe) and lipid values were collected from medical records.

**Results:**

In Austria, 363 patients were enrolled. At baseline, 52% of patients initiated evolocumab without background LLT; the median (Q1, Q3) initial low-density lipoprotein cholesterol (LDL-C) level was 142 (111, 187) mg/dL. Within 3 months of evolocumab treatment, median LDL‑C decreased by 59% to 58 (37, 91) mg/dL. This reduction was maintained over time, despite consistently infrequent use of background LLT. LDL-C < 55 mg/dL was attained by 65% of patients (76% with, 55% without background LLT). Evolocumab persistence was ≥ 90% at month 12 and month 30.

**Conclusion:**

In Austria, patients were initiated on evolocumab at LDL‑C levels almost 3‑times higher than the guideline-recommended clinical goal (< 55 mg/dL). Persistence with evolocumab was very high. Evolocumab led to a rapid and sustained LDL‑C reduction with 65% attaining the LDL‑C goal. Patients using evolocumab in combination with statins and/or ezetimibe were more likely to attain their LDL‑C goal and thus decrease cardiovascular risk.

## Introduction

Cardiovascular disease (CVD) is the most frequent cause of death in Austria, claiming 31,403 lives in 2021 [1]. One of the main risk factors for CVD is high low-density lipoprotein cholesterol (LDL-C), which is associated with increased cardiovascular (CV) events [2]. There is strong evidence that the larger the reductions of LDL‑C, the greater the reduction in the CV event rate [3–5]. Evidence from Mendelian randomization studies suggests that there is no risk in lowering LDL‑C to very low levels [6, 7]. Therefore, it is important to manage elevated LDL‑C levels effectively and early in patients at risk of CVD [3, 8, 9], as shown by the FOURIER open-label extension study [10]. According to the 2019 European Society of Cardiology (ESC)/European Atherosclerosis Society (EAS) guidelines on LDL‑C goals and lipid lowering therapy (LLT), patients with very high CV risk should achieve LDL‑C levels of < 55 mg/dL, and those with high CV risk should achieve < 70 mg/dL, with an LDL‑C reduction by ≥ 50% from baseline or estimated untreated levels in both groups. In individuals with extremely high CV risk, i.e. those with preexisting atherosclerotic cardiovascular disease (ASCVD) who experience a second vascular event within 2 years despite taking maximally tolerated statin therapy, an LDL‑C goal of < 40 mg/dL may be considered [11]. In the past, and prior to the widespread availability of proprotein convertase subtilisin/kexin type 9 inhibitors (PCSK9i), studies consistently demonstrated the difficulty of achieving the recommended goals [12–17]. There is compelling evidence that patients with insufficient LDL‑C reduction need a combination of high-intensity statins and other LLTs [8, 9, 11, 17–19]. Moreover, maintaining attained LDL‑C reductions is important for preventing CV events [20, 21]. LDL‑C stability over time depends on persistence with therapy and on response variability to the administered treatments [22].

The HEYMANS (cHaractEristics of hYperlipidaeMic pAtieNts at initiation of evolocumab and treatment patternS) study [16, 23], showed large and sustained LDL‑C reductions and high persistence to evolocumab on a population level in the real-world setting across 12 European countries. Data from the Austrian HEYMANS cohort are reported here.

## Methods

### Study population and design

HEYMANS (ClinicalTrials.gov Identifier: NCT02770131) was a pan-European, multicenter, observational study enrolling patients who initiated evolocumab as part of their routine clinical management and within local reimbursement criteria. At the time of enrolment, evolocumab was reimbursed in Austria as a secondary prevention in patients with established ASCVD and LDL‑C levels > 100 mg/dL despite maximally tolerated statin therapy and/or intolerance of or contraindication to statins [24].

**Fig. 1:**
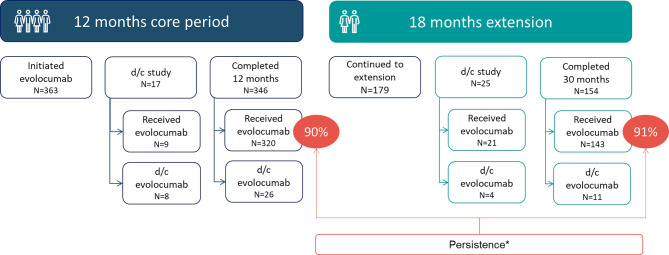
Study periods, patient disposition, and evolocumab persistence. The Month 30 data are based on those still on study at Month 12 (i.e. those who entered the extended follow-up period), and so are not based on the full study population. At month 12, evolocumab status, i.e. continued or stopped, was known for 354 patients and of these, 90% (*n* = 320) were persistent with evolocumab. At month 30, evolocumab status of 158 patients was known and of these, 91% (*n* = 143) were persistent with evolocumab (*d/c* discontinued; *Data are included for patients for whom evolocumab use status could be ascertained at the given time points (i.e. excluding those who ended the study but were still on evolocumab treatment))

Adult patients were included if they received at least one dose of evolocumab after 1 August 2015. Patients who received a PCSK9i as part of an interventional trial or in routine clinical practice within 12 weeks prior to initiation of evolocumab were excluded. Data from 6 months before and up to 30 months after evolocumab initiation were collected. The study consisted of a 12-months core post-baseline observation period. Additionally, a protocol amendment (dated 13 February 2018) allowed for an extension period (month 13 to 30; Fig. 1). Only patients still in the study at the time of approval of the amendment were eligible for the extension period. Austrian study centers collected data between 4 May 2016 (first patient enrolled) and 30 June 2021 (last patient ending observation). Further study details have previously been published by Ray et al. [16, 23].

### Study objectives

The primary objective was to describe the characteristics of patients receiving evolocumab. The secondary objectives were to describe LDL‑C and other lipids and the use of evolocumab and other LLTs over time. Exploratory objectives included estimation of the long-term stability of the attained LDL‑C reductions and treatment results in patients with coronary heart disease (CHD) or a previous myocardial infarction (post-MI).

### Statistical considerations

No formal hypothesis was tested. All analyses were descriptive using summary statistics. Categorical data were presented as frequencies and percentages; continuous data were shown as mean with standard deviation (SD) or median with first (Q1) and third quartile (Q3). When appropriate, 95% confidence intervals (CI) were produced. SAS version 9.4 (SAS Institute, Cary, NC, USA) was used for statistical analysis.

Persistence was defined as the proportion of patients who continued to receive evolocumab and remained in the study at specified time points. Those who stopped the study before these time points but who were still receiving evolocumab were excluded from the persistence analysis. Patients were considered to have discontinued evolocumab if they stopped therapy during the observation period. Evolocumab persistence was analyzed separately for two time periods: 0–12 months and 13–30 months [23].

## Results

### Patient characteristics

Of the 363 patients enrolled in Austria, 61% (*n* = 222) were male. The mean (SD) age was 62 (10.7) years. Most patients (94%, *n* = 342) were in secondary prevention, 73% (*n* = 267) had CHD, 45% (*n* = 164) were post-MI patients. Two-thirds of patients (66%, *n* = 240) had ASCVD without familial hypercholesterolemia (FH), 28% (*n* = 102) had ASCVD with FH, 4% (*n* = 13) had FH without ASCVD, and 2% (*n* = 8) had neither ASCVD nor FH (all 8 patients had high or very high LDL‑C levels and 7 had a history of statin intolerance to between 1 and 3 statins).

Hypertension was present in 73% (*n* = 266) and 32% (*n* = 115) had a diagnosis of FH. A history of statin intolerance was documented for 66% (*n* = 241). Fifty-six percent (*n* = 204) were current or former smokers and 22% (*n* = 80) had diabetes mellitus type 2 (Table 1).

**Table 1:** Patient characteristics at baseline

Baseline characteristic	All patients (*N* = 363)
*Male sex, n (%)*	222 (61)
*Age (years), mean (SD)*	62 (10.7)
*Primary prevention, n (%)*	21 (6)
*Secondary prevention, n (%)*	342 (94)
Coronary heart disease	267 (73)
Post-myocardial infarction	164 (45)
*Familial hypercholesterolemia, n (%)*	115 (32)
*Type 2 diabetes, n (%)*	80 (22)
*Hypertension, n (%)*	266 (73)
*Chronic kidney disease, n (%)*	31 (9)
*Prior/current smokers, n (%)*	204 (56)
*History of intolerance to any statin, n (%)*	241 (66)

### Use of evolocumab and background LLTs over time

The baseline evolocumab dose was 140 mg once every two weeks in most patients (99%, *n* = 361). At month 12, evolocumab status, i.e. continued or stopped, was known for 354 patients, 90% of which (*n* = 320) were persistent with evolocumab. In the extension phase (month 13–30), 179 patients continued to be followed. Evolocumab status at month 30 was known for 158 patients. Thereof, 91% (*n* = 143) were persistent (Fig. 1).

**Fig. 2:**
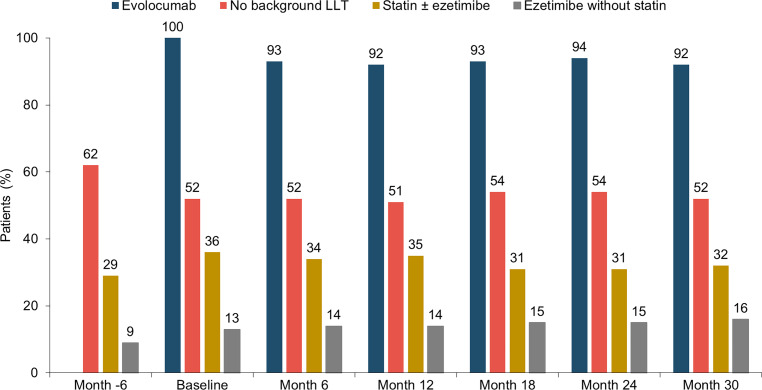
Dyslipidemia management patterns over time. Background LLT = statin ± ezetimibe (*LLT* lipid-lowering therapy)

At baseline, 52% (*n* = 187) received evolocumab without background LLT, 36% (*n* = 129) received evolocumab in combination with statins ± ezetimibe, and 13% (*n* = 47) received evolocumab in combination with ezetimibe. Use of background LLT was similar in women (48%, *n* = 68/141) and men (49%, *n* = 108/222). Of patients receiving statins at baseline, 72% (*n* = 93/129) received a high-intensity and 22% (*n* = 28/129) a moderate-intensity statin. Women were less likely to receive a high-intensity statin (22% [31/141] versus 28% [62/222]) or statins in combination with ezetimibe (19% [27/141] versus 22% [49/222]). Patterns of background LLT remained stable over time (Fig. 2).

### LDL-C levels over time and goal attainment

**Fig. 3:**
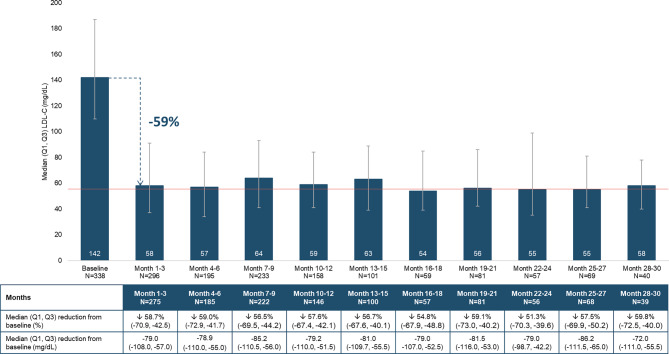
LDL‑C over time (*LDL‑C* low-density lipoprotein cholesterol; *Q* quartile)

**Fig. 4:**
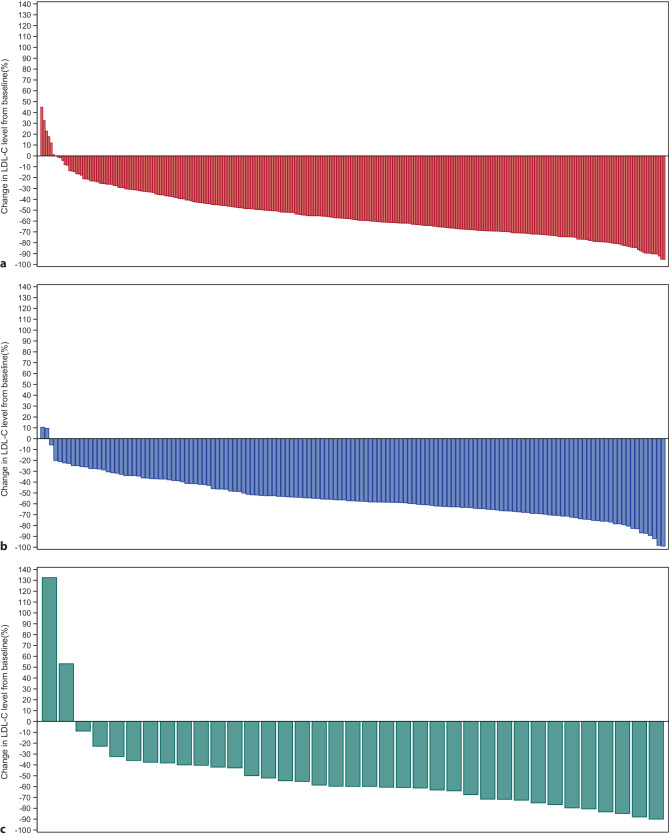
LDL‑C variability outcomes. **a** Months 1 to 3, N = 268. **b** Months 10 to 12, N = 143. **c** Months 28 to 30, N = 37. Figures (**a**) to (**c**) show on-treatment percentage change in LDL‑C for patients with baseline LDL–C ≥ 70 mg/dL (*LDL‑C* low-density lipoprotein cholesterol)

The median (Q1, Q3) baseline LDL‑C was 142 (111, 187) mg/dL overall and was lower in patients with background LLT at baseline (127 [102, 161] mg/dL) versus those without (161 [123, 197] mg/dL). Baseline LDL‑C was higher in women (166 [123, 202] mg/dL) than men (129 [103, 167] mg/dL). Within the first 3 months of evolocumab therapy, the median (Q1, Q3) LDL‑C level was reduced by 59% to 58 (37, 91) mg/dL (Fig. 3). The percentage reduction in on-treatment LDL‑C among patients with a baseline LDL-C ≥ 70 mg/dL remained consistent over time at months 1–3, 10–12, and 28–30 (Fig. 4).

**Fig. 5:**
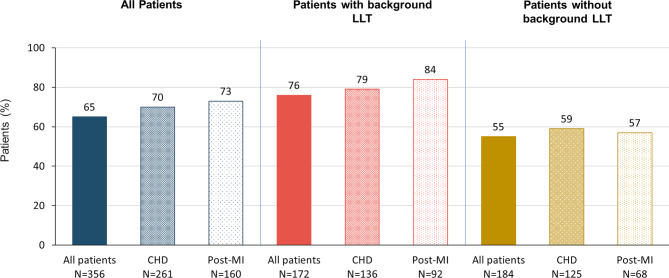
Attainment of LDL-C < 55 mg/dL. The 2019 ESC/EAS guidelines [11] recommend a ≥ 50% LDL‑C reduction and the achievement of < 55 mg/dL for patients with very high cardiovascular risk and LDL-C < 70 mg/dL for patients with high cardiovascular risk. LDL‑C goal attainment data are missing for 7 patients. In the Austrian cohort, 351 patients had very high and 11 patients had high cardiovascular risk (1 patient had neither) (*CHD* coronary heart disease; *LDL‑C* low-density lipoprotein cholesterol; *LLT* lipid-lowering therapy; *post-MI* post myocardial infarction)

**Fig. 6:**
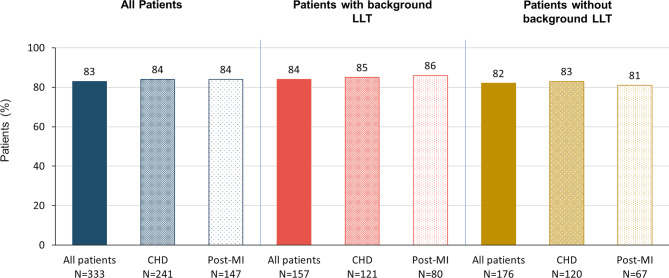
Attainment of an LDL‑C reduction of ≥ 50% from baseline. The 2019 ESC/EAS guidelines [11] recommend a ≥ 50% LDL‑C reduction and the achievement of LDL-C < 55 mg/dL for patients with very high and < 70 mg/dL for patients with high cardiovascular risk. LDL‑C goal attainment data are missing for 7 patients. In the Austrian cohort, 351 patients had very high and 11 patients had high cardiovascular risk (1 patient had neither) (*CHD* coronary heart disease; *LDL‑C* low-density lipoprotein cholesterol; *LLT* lipid-lowering therapy; *post-MI* post myocardial infarction)

Among patients with at least one post-baseline LDL‑C measurement, 65% (*n* = 232/356) achieved the LDL‑C goal of < 55 mg/dL (CHD: 70%, *n* = 182/261; post-MI: 73%, *n* = 116/160). Attainment of LDL‑C goals was higher among patients receiving background LLT (all patients: 76%, *n* = 131/172; CHD: 79%, *n* = 108/136; post-MI: 84%, *n* = 77/92) compared to those receiving evolocumab alone (all patients: 55%; *n* = 101/184; CHD: 59%, *n* = 74/125; post-MI: 57%, *n* = 39/68; Fig. 5). Attainment of LDL-C < 55 mg/dL was 50% (*n* = 69/138) in women and 75% (*n* = 163/218) in men. Between 81 and 86% of patients attained a ≥ 50% LDL‑C reduction across all groups (Fig. 6).

### Safety

Forty-eight patients (13%) reported adverse drug reactions (ADRs); the most frequent were musculoskeletal and connective tissue disorders (7%, *n* = 26). Two ADRs were considered serious (acute MI, angina pectoris). Both patients had a history of severe CVD and were at extremely high risk of recurrent events. The outcome of both ADRs were reported as resolved and neither patient discontinued evolocumab. No fatal ADRs occurred (Table 2).

**Table 2:** Treatment-emergent adverse drug reactions to evolocumab

System Organ Class Preferred Term	All patients *N* = 363
**Adverse drug reactions, *n* (%)**	48 (13)
*Cardiac disorders, n (%)*	2 (1)
Acute myocardial infarction	1 (< 1)
Angina pectoris	1 (< 1)
*Ear and labyrinth disorders, n (%)*	2 (1)
Vertigo	2 (1)
*Gastrointestinal disorders, n (%)*	6 (2)
Abdominal discomfort	1 (< 1)
Abdominal pain upper	1 (< 1)
Constipation	2 (1)
Diarrhea	1 (< 1)
Nausea	1 (< 1)
*General disorders and administration site conditions, n (%)*	13 (4)
Asthenia	2 (1)
Discomfort	1 (< 1)
Fatigue	3 (1)
Influenza-like illness	1 (< 1)
Injection site hematoma	3 (1)
Injection site rash	2 (1)
Edema peripheral	1 (< 1)
Sensitivity to weather change	1 (< 1)
*Infections and infestations, n (%)*	3 (1)
Bronchitis	1 (< 1)
Rhinitis	2 (1)
Viral infection	1 (< 1)
*Investigations, n (%)*	3 (1)
Blood creatinine phosphokinase increased	1 (< 1)
Blood triglycerides increased	1 (< 1)
Hepatic enzyme abnormal	1 (< 1)
*Metabolism and nutrition disorders, n (%)*	1 (< 1)
Type 2 diabetes mellitus	1 (< 1)
*Musculoskeletal and connective tissue disorders, n (%)*	26 (7)
Arthralgia	6 (2)
Back pain	2 (1)
Bone pain	2 (1)
Limb discomfort	1 (< 1)
Muscle spasms	1 (< 1)
Muscle weakness	1 (< 1)
Myalgia	13 (4)
Myositis	1 (< 1)
Osteitis	1 (< 1)
Pain in extremity	1 (< 1)
*Nervous system disorders, n (%)*	6 (2)
Disturbance in attention	1 (< 1)
Dizziness	2 (1)
Headache	4 (1)
Memory impairment	1 (< 1)
Paresthesia	1 (< 1)
*Respiratory, thoracic and mediastinal disorders, n (%)*	2 (1)
Oropharyngeal pain	1 (< 1)
Rhinorrhea	1 (< 1)
*Skin and subcutaneous tissue disorders, n (%)*	6 (2)
Alopecia	1 (< 1)
Erythema	1 (< 1)
Pruritus	1 (< 1)
Rash	2 (1)
Urticaria	1 (< 1)
*Vascular disorders, n (%)*	1 (< 1)
Flushing	1 (< 1)
**Serious adverse drug reactions, *n* (%)**	**2 (1)**
*Cardiac disorders, n (%)*	2 (1)
Acute myocardial infarction	1 (< 1)
Angina pectoris	1 (< 1)

## Discussion

Previous studies have consistently demonstrated inadequate LDL‑C goal attainment across regions and healthcare settings. LDL‑C goal attainment mainly depends on two factors: the severity of dyslipidemia and the treatment intensity. Treatment intensity not only depends on the prescribed medication but also on adherence to treatment. In the Austrian HEYMANS cohort, the median baseline LDL‑C was 142 mg/dL, which was reduced by 59% within the first 3 months of evolocumab therapy. Sixty-five percent achieved the LDL‑C goal of < 55 mg/dL. Patients receiving evolocumab with background LLT had higher levels of LDL‑C goal attainment (76% versus 55%, respectively).

In Austria, patients received evolocumab mostly in secondary prevention (i.e. after they had already experienced a CV event). This is in accordance with the Austrian reimbursement regulations, which restricts PCSK9i use to secondary prevention in patients on maximally tolerated statin/ezetimibe therapy. At the onset of the study, reimbursement regulations further required patients to meet certain LDL‑C levels before (> 100 mg/dL) and during treatment (target of < 70 mg/dL), based on the 2016 ESC/EAS dyslipidemia guidelines in force at the time [25]. During the enrolment period, initial PCSK9i prescriptions and thus high-intensity combination therapy, could only be provided in 27 specialized centers of endocrinology and metabolic diseases appointed by the Austrian Federation of Social Insurances [24]. However, in July 2022, after this study was completed, the Austrian health authorities allowed more specialties (cardiologists and neurologists) to prescribe evolocumab, the numbers of appointed centers increased to 100, and the treatment reimbursement threshold was lowered to > 70 mg/dL with an on-treatment target of < 55 mg/dL [26], as recommended by the 2019 ESC/EAS guidelines [11]. These changes represent an important step to improve access to intensified LLT for patients at very high CV risk in Austria.

The DA VINCI study was a multinational survey that examined how well patients with high or very high CV risk achieved their LDL‑C goals. The study was conducted before PCSK9i were widely used (only 1% used a PCSK9i in DA VINCI). In the Austrian DA VINCI cohort, only 38% reached their risk-based LDL‑C goals, which were < 55 mg/dL for very high CV risk and < 70 mg/dL for high CV risk. Among secondary prevention patients, only 23% attained LDL-C < 55 mg/dL, despite receiving high-intensity statins (45%), moderate-intensity statins (34%) or a statin-ezetimibe combination (14%) as the most frequently prescribed LLTs [12]. While there were some differences in goal achievement between men (75%) and women (50%) in Austria, it should be noted that women had a higher baseline LDL‑C and were less likely to receive a high-intensity statin or combination with ezetimibe. In the Austrian DA VINCI cohort [12], women were also less likely to attain their LDL‑C goals. In the FOURIER trial, LDL‑C reductions were greater in men (58%) than women (52%, *p* < 0.001) but absolute CV risk reduction was similar [27].

In HEYMANS Austria, approximately two-thirds (66%) received evolocumab after they were found to be intolerant to at least one statin; 94% of patients were in secondary prevention. The findings from HEYMANS indicate that adding evolocumab to the LLT regimen increases the proportion of secondary prevention patients who achieve their LDL‑C goal of < 55 mg/dL. The 65% of patients who attained this goal in HEYMANS marks a vast improvement over the 23% in secondary prevention in DA VINCI Austria [12]. The overall DA VINCI study, which included 18 countries and 5888 patients, allowed identification of key clinical insights, such as the importance of highly intensive combination therapy to attain recommended LDL‑C goals [17]. The HEYMANS study confirms these insights from DA VINCI that intensive combination therapy is necessary to reach recommended LDL‑C levels.

In the Austrian HEYMANS cohort, more than half of patients did not receive a statin and/or ezetimibe. The Austrian reimbursement criteria mandate documentation of indicators of statin intolerance, defined as therapeutic attempts with more than one statin (at minimum atorvastatin and rosuvastatin) which led to myopathies and exceeding creatinine kinase normal values by at least 5 times or occurrence of a severe hepatopathy [24]. In the study setting, where all patients were treated in PCSK9i centers with extensive knowledge and experience in dyslipidemia management, it must be assumed that patients received the best possible treatment combination in their respective circumstance of very high CV risk and often combined with statin intolerance.

As stated above, the LDL‑C level at treatment initiation is an important factor in achieving recommended LDL‑C goals. The higher the initial LDL‑C level, the more difficult it becomes to reach LDL-C < 55 mg/dL, which is recommended for very high-risk patients. This leads to the question of why physicians did not refer patients to specialized PCSK9i centers for initiation of highly intensive combination therapy as early as possible when they were not attaining their LDL‑C goals despite taking statins. In clinical practice, individuals with high LDL‑C levels are often first identified in primary care. General practitioners initiate statins as a first step to lower lipid levels and advise on life-style changes. It can be assumed that referrals to specialized clinics often involve only those patients who have LDL‑C levels farthest away from the goal (i.e. those with the highest LDL‑C levels). This is supported by the finding that most patients in our analysis had no background LLT before (62%) or at the start of evolocumab (52%). In patients not receiving background LLT at baseline, LDL‑C was substantially higher than in those with background LLT, which can be considered a contributor to the lower goal attainment in these patients. As evolocumab was relatively new at the time of the study, the population enrolled may be biased towards patients for whom there was no adequate treatment as the time. Across the countries participating in HEYMANS, it was consistently found that patients were often started on evolocumab when their LDL‑C levels were above the threshold set by the insurers or the government [16]. In addition to the barriers within general practice, patients released from hospitals after an acute vascular event and receiving an initial LLT prescribed by the hospital, are not always adequately followed-up to monitor if they have reached their recommended target LDL‑C levels. Therefore, their LLT might not be intensified as needed. It is therefore essential to raise awareness on the importance of starting intensive LLT as soon as possible after a vascular event, ideally high-intensity statins ± ezetimibe with intensification using PCSK9is. For patients who cannot tolerate statins and are receiving evolocumab monotherapy, additional options for treatment intensification could include low dose statins, ezetimibe, bempedoic acid, or a combination of bempedoic acid and ezetimibe.

This study has some limitations. In Austria, recruitment started in May 2016 when PCSK9is were relatively newly reimbursed. Therefore, the patients selected for evolocumab treatment and subsequently enrolled into this study may have been those who had persistently high LDL‑C levels despite previous therapies. A selection of more difficult-to-treat patients may have impacted on the findings. Evolocumab persistence may have been slightly overestimated by two factors: patients who were receiving evolocumab for a longer period before enrolment (maximum 6 months) may have been more likely to continue with their treatment than those who initiated treatment closer to the enrolment date. The centers participating in the HEYMANS study were the first to prescribe PCSK9is and had high expertise in all aspects of dyslipidemia management. Such specialists may have been more likely to motivate patients to continue with treatment and to monitor them more closely than a typical scenario in primary care. Nevertheless, our results are similar to those of previous studies on evolocumab persistence [23, 28, 29] and adherence [30]. The study protocol was amended to extend the observation period from 12 to 30 months. Some study centers declined to participate in the extension phase. However, treatment centers in Austria generally follow the same therapeutic principles. Therefore, possible bias resulting from the discontinuation of some study centers is unlikely. Finally, all observational studies carry a risk of potential data misclassification.

## Conclusions

In the Austrian HEYMANS cohort, evolocumab initiation was associated with rapid and sustained reductions in LDL‑C levels. Long-term persistence with evolocumab treatment was very high. Most patients (65%) achieved their LDL‑C goals with evolocumab, and attainment was higher in those who received combination therapy (76%) compared to patients receiving evolocumab alone (55%). Our findings suggest that optimal LLT should be initiated as early as possible, and that regular long-term monitoring is essential. Highly intensive combination therapies including PCSK9i are needed to attain the recommended low levels of LDL‑C.
